# Iatrogenic Obstructive Sialadenitis of the Parotid and Submandibular Glands Following Facial Rejuvenation

**DOI:** 10.1007/s00266-026-05841-z

**Published:** 2026-05-14

**Authors:** Nir Tsur, Ofir Zavdy, Dafna Shilo Yaacobi, Gal Avishai, Uri Alkan, Thomas Shpitzer, Hanna Gilat

**Affiliations:** 1 Department of Otolaryngology-Head and Neck Surgery, Rabin Medical Center—Beilinson Hospital, 39 Jabotinski St., 4941492 Petach Tikva, Israel; 2 Faculty of Medicine (N.T., O.Z., D.Y., S.M., H.G.), Tel Aviv University, Tel Aviv, Israel; 3 Department of Plastic and Reconstructive Surgery, Rabin Medical Center, Sackler Faculty of Medicine, Tel-Aviv University, Tel Aviv, Israel; 4 Department of Oral and Maxillofacial Surgery, Rabin Medical Center, Petach Tikva, Israel; 5 The Maurice and Gabriela Goldschleger School of Dental Medicine, Tel-Aviv University, 69978 Tel Aviv, Israel

**Keywords:** Facial rejuvenation, Obstructive sialadenitis, Sialendoscopy

## Abstract

**Objective:**

During facial rejuvenation procedures, the salivary glands and their ducts are susceptible to injury. Given the paucity of data on obstructive salivary gland complications after facial rejuvenation procedures, this study aimed to characterize their clinical features and assess the outcomes of endoscopic management.

**Methods:**

A tertiary salivary gland clinic database was retrospectively reviewed for patients who presented with obstructive sialadenitis involving the parotid and submandibular glands following facial rejuvenation procedures and were treated with sialendoscopy. Demographic, clinical, and outcome data were collected from the files.

**Results:**

The cohort included 11 patients (8 women, 3 men) with a mean age of 59±5.1 years. The mean follow-up time was two years. Patients presented with gland swelling, pain, xerostomia, hypersalivation, and pruritus. Physical examination indicated parotid involvement in 8 cases and submandibular gland involvement in 3. Preoperative imaging studies revealed gland swelling, ductal system dilation, and ductal strictures. A convoluted and atrophic ductal system with cloudy sediments was frequently observed during surgery. Most patients required multiple sialendoscopic procedures. While the majority were successfully treated endoscopically, one patient required adjunctive open repair for a persistent parotid cutaneous fistula using a sternocleidomastoid muscle flap.

**Conclusions:**

This study presents the most comprehensive case series on obstructive sialadenitis of the parotid and submandibular glands following FRPs. These complications may result in significant morbidity. Sialendoscopy is an effective treatment for these conditions. Proper clinical assessment in the office and operating room is crucial for accurate diagnosis and effective treatment.

**Level of Evidence IV:**

This journal requires that authors assign a level of evidence to each article. For a full description of these Evidence-Based Medicine ratings, please refer to the Table of Contents or the online Instructions to Authors www.springer.com/00266.

## Introduction

The market size for facial rejuvenation procedures (FRPs), both surgical and nonsurgical, has grown globally over the past few decades. In 2018 alone, more than 1,500,000 surgical FRPs and over 3 million nonsurgical FRPs were reported by the American Society for Aesthetic and Plastic Surgery (ASAPS). [1] Nonsurgical minimally invasive facial rejuvenation techniques include barbed sutures (threads), laser or ablation, and injection of botulinum toxin, collagen, and other fillers. [2, 3] Surgical interventions range from classic rhytidectomy to minimal incision facelifts.

The parotid is the largest of the three major paired salivary glands and accounts for 25% of daily saliva secretion. As illustrated in Fig. 2, the parotid glands lie superficial to the masseter and deep to the superficial musculoaponeurotic system (SMAS). Their ducts, extending from the anterior portion of the gland, traverse the masseter muscles before penetrating the buccinator muscle to enter the oral cavity. [3] The glands' proximity to the SMAS makes them susceptible to injuries during rhytidectomy. [3] Saliva from the submandibular glands (SMG), the second-largest paired salivary gland, is transported via Wharton's duct, which curves around the posterior border of the mylohyoid muscle. [4]

Although there are several reports of parotid and SMG Injuries following FRPs, most involve subcutaneous sialoceles and fistulas due to capsular interruption. [5–8] Fewer than 40 cases of iatrogenic parotid injuries after rhydectomy have been documented, and information specifically on obstructive glandular injuries following FRPs remains limited. This study aimed to report a series of 11 patients with chronic obstructive parotitis or SMG sialadenitis without sialolithiasis following FRPs.

## Materials and Methods

The local Institutional Review Board approved the study. The electronic database of the Salivary Gland Clinic at a tertiary medical center (2016 to 2021) was retrospectively searched for patients aged 18 years or older who were diagnosed with obstructive parotitis or sialadenitis following FRPs and were subsequently assessed and treated with sialendoscopy. Inclusion criteria comprised patients with endoscopic or imaging evidence of non-anatomic ductal obstruction following FRPs, without other identifiable etiologies (e.g., sialolithiasis, autoimmune disease, infection). Exclusion criteria included sialoceles, mucoceles, postoperative fistulae, or prior head and neck irradiation. Imaging interpretation was performed by an experienced head and neck radiologist blinded to clinical outcomes. Data on demographic characteristics, medical history, clinical findings, treatment, and outcomes were collected for all eligible patients. Facial rejuvenation procedures included either deep-plane rhytidectomy with or without SMAS plication (*n* = 9) or thread-lift procedures using polydioxanone sutures (*n* = 2), as confirmed by operative reports or patient interviews when available.

In cases of ductal stenosis, a 0.9-mm semi-rigid sialendoscope was advanced across the narrowed segment following sequential papillary dilation using the Seldinger technique. Ductal strictures were managed with graded mechanical dilation and blunt endoscopic passage, followed by copious saline irrigation. Intraductal steroid instillation (20 mg dexamethasone) was performed to reduce periductal inflammation. Mucous plugs or foreign material, when present, were gently mobilized and flushed until a freely patent lumen was visualized'. Data were analyzed with descriptive statistics.

## Results

The cohort consisted of 11 patients with obstructive parotitis or SMG sialadenitis following FRPs. Their details are shown in Table 1: eight women (73%) and three men of mean age 59±5.1 years (range 27–73). Underlying medical conditions included diabetes mellitus type 1 in one patient and cerebral vascular accident and hypothyroidism in another. Nine patients (82%) had undergone deep-plane rhytidectomy, and two (18%) had undergone facial thread procedures. No patient reported acute postoperative sialadenitis, sialocele, or salivary fistula in the immediate postoperative period; all symptoms developed after a prolonged asymptomatic interval. The average time from the FRP to symptom onset was 5.1±1.8 years (range 2–20), and the average duration of follow-up after diagnosis of obstructive sialadenitis was 2.3±0.6 years.

**Table 1:** Patient characteristics and outcomes in post-facial rejuvenation procedures with obstructive sialadenitis

Pt. No.	Sex/ age (yr)		Medical history	FRP		Time lapsed		Gland injured		Side		Symptoms		Physical examination		Imaging		Sialendoscopy		Treatments		No. endoscopies
1	M/65		N/A	Facelift		10 y		P		Bilateral		Swelling, xerostomia		Enlarged glands, viscous saliva, and sediments		CT sialography-diffuse constriction of Stensen		Severe gland enlargement, convoluted ductal system, several areas of constriction, viscous saliva		Vigorous rinsing, steroids, antibiotics		5
2	M/67		Smoker	Facelift		2 y		P		Left		Swelling		Enlarged gland, hyposalivation		Normal CT		Severe gland enlargement, convoluted ductal system, several areas of constriction		Vigorous rinsing, steroids, antibiotics		3
3	F/72		N/A	Threads		2 y		SMG		Right		Swelling, pain		Hypersalivation, tenderness		Normal CT		Thread coursing the gland		Vigorous rinsing, steroids, antibiotics		2
4	F/69		Diabetes	Facelift		10 y		SMG		Left		Swelling		Enlarged gland		US- Enlarged SMG ductal system, hypoechoic		Severe gland deformity, unable to reach hilum		Vigorous rinsing, steroids, antibiotics		2
5	F/27		N/A	Threads		5 y		SMG		Bilateral		Swelling, pain		Diffuse, bilateral enlargement, hypersalivation		N/A		Severe ductal constrictions in multiple areas		Vigorous rinsing, steroids, antibiotics		1
6	F/66		N/A	Facelift		4 y		P		Left		Swelling, xerostomia		Enlarged gland, hyposalivation		Normal CT		Convoluted fibrous duct, constricted in proximal and distal areas		Vigorous rinsing, steroids, antibiotics		3
7	F/72	Smoker			Facelift		2 y		P		Bilateral		Swelling, xerostomia		Enlarged glands, viscous saliva, and sediments		Normal CT		Severe gland enlargement, convoluted ductal system, several areas of constriction		Vigorous rinsing, steroids, antibiotics	1
8	M/62	N/A			Facelift		2 y		P		Left		Swelling, xerostomia		Enlarged glands, viscous saliva, and sediments		Normal CT		Severe ductal constrictions in multiple areas, viscous sediments		Vigorous rinsing, steroids, antibiotics	2
9	F/27	N/A			Facelift		5 y		P		Bilateral		Swelling, xerostomia, pruritis		Enlarged glands, viscous saliva, and sediments		US- enlarged Stensen duct		Severe ductal constrictions in multiple areas, viscous sediments		Vigorous rinsing, steroids, antibiotics	2
10	F/73	N/A			Facelift		20 y		P		Left		Swelling, pain		Enlarged gland, good salivation		N/A		Single proximal occlusion of Stensen’s duct, unable to pass the endoscope		Vigorous rinsing, steroids, antibiotics	1
11	F/63	CVA, hypothyroidism, celiac, Frey's syndrome			Facelift		2 y		P		Left		Recurrent swelling		Salivary discharge via cutaneous fistula		CT sialography-severe midpart and proximal ductal (Stensen) dilation, and parotid cutaneous fistula		Multiple strictures and a single distal occlusion (kink)		Vigorous rinsing, steroids, antibiotics	1

*CT* Computed tomography, *CVA* Cerebrovascular accident, *FRP* Facial rejuvenation procedure, P Parotid gland, *SMG* Submandibular gland, *syndr* Syndrome, *US* Ultrasound

Symptoms included gland swelling (100%), pain (27%), xerostomia (45%), hypersalivation (18%), and pruritus (9%). Physical examination revealed parotid involvement in eight cases (73%) and SMG involvement in three (27%). Seven patients (64%) had unilateral sialadenitis, and four (36%) had bilateral disease.

**Fig. 1:**
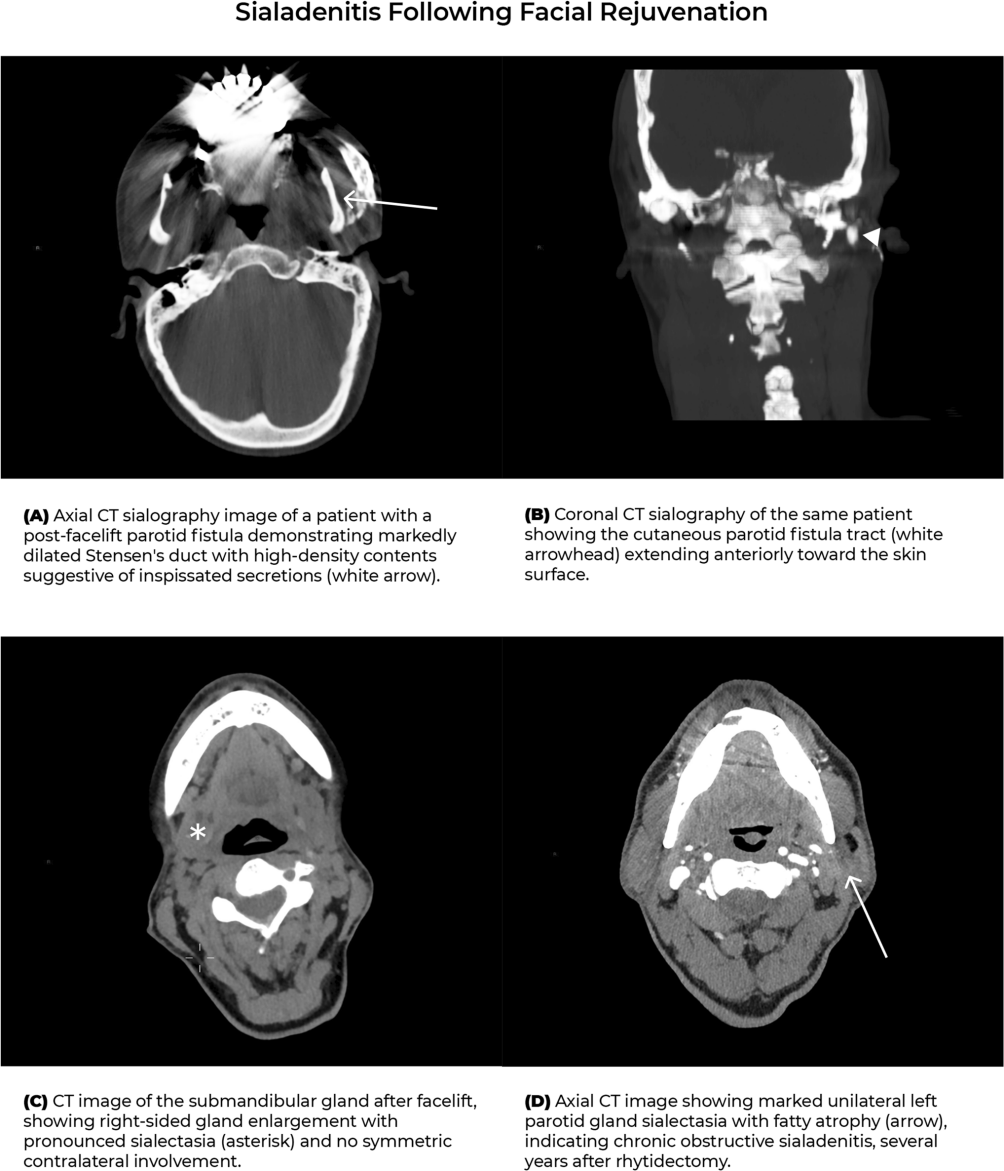
Representative radiologic and sialographic findings in patients with obstructive sialadenitis after facial rejuvenation procedures. **A**. Sialography showing markedly dilated left Stensen’s duct with turbid content and heavy sediment. **B**. Sialography showing contrast extravasation through a cutaneous fistula (arrow), confirming sialocutaneous tract formation. **C**. Axial CT image showing enlarged right submandibular gland with sialoectasia (arrowhead). **D**. Coronal CT confirming left-sided atrophy and asymmetric ductal disease (arrow)

Imaging studies (computed tomography, computed tomography sialography, ultrasonography) showed gland swelling, ductal system dilation, and ductal strictures in four patients (Table 1). Representative images are presented in Fig. 1.

**Fig. 2:**
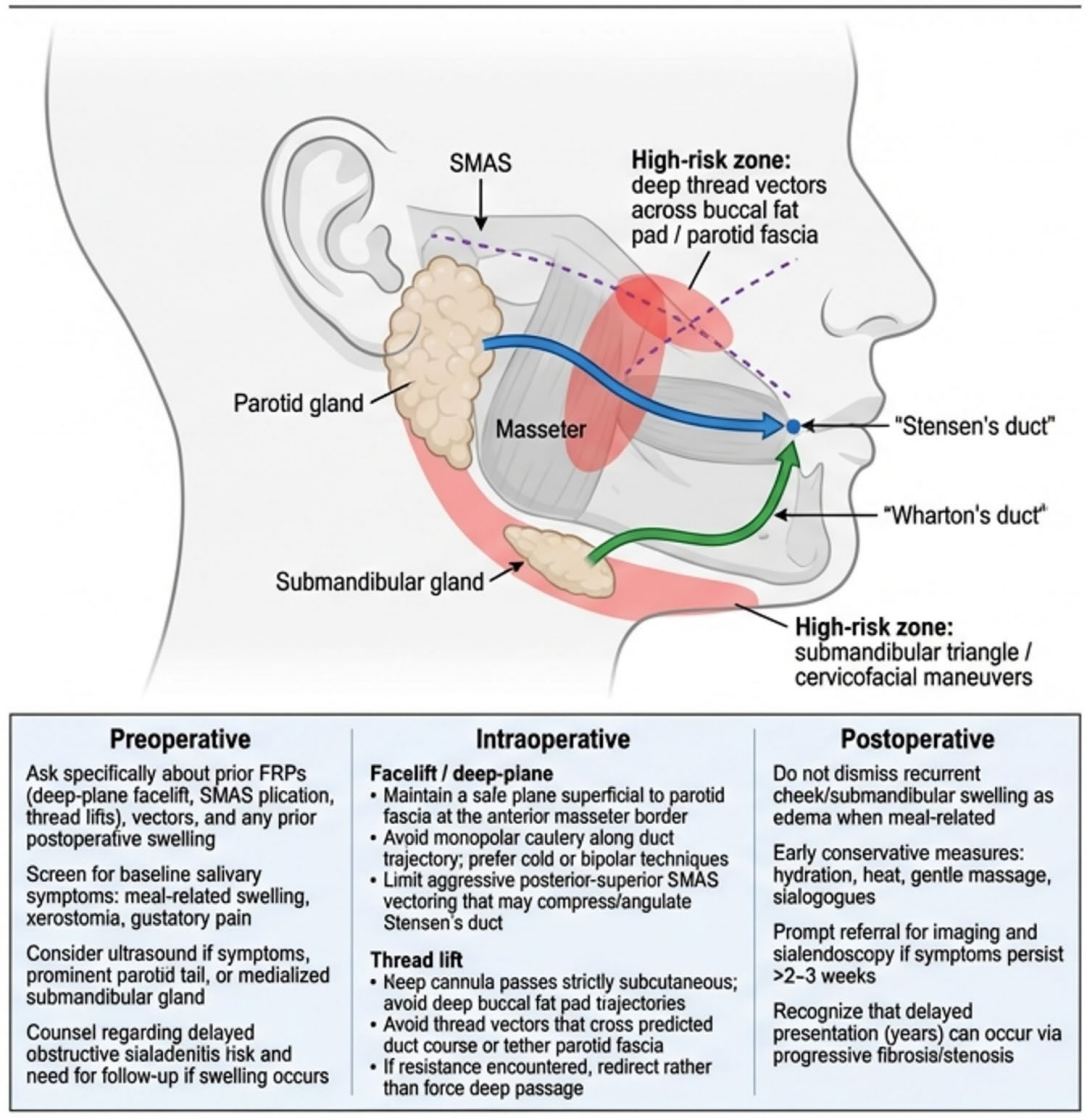
Anatomical course of Stensen’s and Wharton’s ducts highlighting high-risk dissection zones during facial rejuvenation procedures. The lower panel outlines key preventive recommendations across the preoperative, intraoperative, and postoperative phases to reduce the risk of obstructive sialadenitis, summarizing phase-specific pitfalls and strategies derived from our series and the salivary obstruction literature

All patients underwent sialendoscopic management. Stensen’s duct was affected in eight patients, and Wharton’s duct in 3. Endoscopic findings included intraductal strictures (*n* = 7), mucous plugging (*n* = 6), and convoluted or scarred ductal pathways (*n* = 5). In one patient, a foreign body consistent with a retained thread was visualized at the ductal orifice and removed endoscopically. No transections, thermal burns, or active sialoceles were identified. All patients received saline irrigation, ductal dilation, and steroid instillation (Fig. 2).

Seven patients (63%) required additional sialendoscopies under either local (45%) or general (55%) anesthesia to treat obstructive symptoms, for an average of 2.1 endoscopies per patient (range 1–5). Adjunctive treatments included vigorous rinsing, steroid administration, and antibiotics. More pervasive and repeated interventions were required in patients with bilateral gland involvement, severe ductal restriction, or extensive gland deformities with convoluted ductal systems. One patient presented with a persistent parotid cutaneous fistula and required adjunctive open surgical repair with placement of a sternocleidomastoid (SCM) muscle flap following multiple endoscopic sessions that achieved significant ductal improvement. Despite the complexity of these cases, all patients eventually achieved relief from swelling, pain, and xerostomia with the combination of sialendoscopic procedures and adjunctive treatments. In two patients with persistent postoperative gland prominence and discomfort without active ductal obstruction, ultrasound-guided botulinum toxin type A injections were administered (20–30 units for the parotid gland or 15–20 units for the submandibular gland, divided into 3–4 intraparenchymal aliquots). Both patients reported improvement in gland fullness and tension without clinically significant xerostomia.

## Discussion

Chronic obstructive sialadenitis is characterized by impaired salivary outflow due to ductal narrowing or distortion, resulting in recurrent gland swelling, pain, and progressive inflammation. Common etiologies include sialolithiasis, congenital or acquired ductal anomalies, autoimmune disease, infection, and iatrogenic injury [9]. In recent years, increasing attention has been directed toward iatrogenic causes related to head and neck procedures, including facial rejuvenation interventions.

Parotid-related complications following rhytidectomy are classically described as sialocele or salivary fistula resulting from capsular violation [10, 11]. In contrast, obstructive salivary gland disease following facial rejuvenation procedures remains infrequently reported and is likely underrecognized. Prior studies describing complications of facial rejuvenation have largely focused on hematoma, infection, nerve injury, and aesthetic outcomes, with limited attention to delayed ductal obstruction or chronic sialadenitis [12, 13]. Our series therefore expands the current literature by characterizing a distinct pattern of delayed, non-lithiasic obstructive salivary gland disease following facial rejuvenation procedures.

The proposed pathophysiologic mechanism involves ductal distortion, compression, or scarring rather than acute transection. In deep-plane rhytidectomy, elevation and manipulation of the SMAS over the anterior masseter and parotid capsule may impart traction, compression, or thermal injury along the course of Stensen’s duct, predisposing to delayed stricture formation [5, 6, 14, 15]. In thread-lift procedures, deep cannula passes or barbed suture vectors traversing the buccal fat pad or parotid fascia may result in focal ductal kinking or tethering. These technique-specific mechanisms likely explain the localized ductal stenoses observed in our cohort.

Postoperative gland prominence and contour changes represent a related but distinct phenomenon. Azizzadeh et al. reported that cervicofacial rejuvenation procedures may accentuate submandibular gland visibility in up to 29% of patients due to SMAS tightening, platysmal manipulation, and reduced soft tissue masking [7, 16]. In selected patients with symptomatic gland deformity or fullness following facial rejuvenation procedures, chemodenervation with botulinum toxin may reduce salivary flow and gland turgor, thereby improving contour and discomfort while avoiding gland excision. In our practice, this approach is reserved for patients without active ductal obstruction and is performed using the lowest effective dose, with appropriate counseling regarding transient xerostomia and the potential need for repeat injections [8]

Although a direct causal relationship between facial rejuvenation procedures and obstructive sialadenitis cannot be established in this small, retrospective, referral-based cohort, several consistent observations support an association. Ductal pathology occurred exclusively in glands anatomically adjacent to prior surgical or thread-lift dissection fields, alternative etiologies were excluded, and unilateral or asymmetric involvement predominated. Importantly, the prolonged latency to symptom onset observed in this series suggests a chronic, progressive fibrotic process rather than an acute ductal injury. These findings should therefore be interpreted as hypothesis-generating and do not permit estimation of absolute risk or definitive causality. Accordingly, the primary purpose of this study is not to establish incidence or causality, but rather to raise awareness among facial plastic surgeons of a rare, delayed, and potentially preventable complication, and to emphasize the importance of anatomical vigilance, early recognition, and minimally invasive management.

Delayed presentation is a key clinical feature of this entity. The gradual development of periductal fibrosis may remain clinically silent for years before progressing to functionally significant obstruction, consistent with prior descriptions of delayed salivary duct strictures following facial procedures [17] This prolonged asymptomatic interval likely contributes to underdiagnosis and delayed referral.

Sialendoscopy is now well established as a minimally invasive approach for the diagnosis and management of obstructive salivary gland disorders, including ductal stenosis and chronic non-lithiasic sialadenitis [18–21]. Endoscopic dilation, irrigation, and targeted intraductal therapy can restore salivary flow, reduce inflammation, and preserve gland function, particularly when intervention is performed before advanced fibrosis develops [20, 22–29]. In the present cohort, sialendoscopy enabled effective symptom control while avoiding gland excision, although repeated interventions were required in patients with advanced or bilateral disease.

From a surgeon’s perspective, prevention remains paramount. Preoperative assessment should include targeted questioning regarding prior facial rejuvenation procedures and any history of salivary gland symptoms. Intraoperatively, maintaining a safe dissection plane superficial to the parotid fascia at the anterior masseteric border, avoiding monopolar cautery along the ductal trajectory, and limiting aggressive SMAS vectoring may reduce the risk of ductal distortion. During thread-lift procedures, strictly subcutaneous cannula passes and avoidance of deep vectors across the buccal fat pad or parotid fascia are essential. Phase-specific pitfalls and preventive strategies derived from our series and the salivary obstruction literature are summarized in Table 2. [30–33]

**Table 2:** Surgical phase–specific pitfalls and preventive strategies

Surgical phase	Potential pitfalls	Preventive strategies
Preoperative	Prior rhytidectomy scarring Altered SMAS from previous cosmetic interventions Prominent parotid tail or medialized SMG increasing vulnerability Unrecognized baseline ductal stenosis	Assess baseline salivary gland function Review prior surgeries and thread paths Consider ultrasound if symptoms or prior complications Preoperative counseling on risk
Intraoperative (Facelift)	Dissection too deep over anterior masseter Traction injury from aggressive SMAS elevation Thermal injury from monopolar cautery over buccal fat pad Excessive posterior–superior SMAS tension	Maintain superficial dissection plane Use cold or bipolar cautery near duct course Gentle SMAS mobilization Avoid over-tight vectoring that compresses parotid duct
Intraoperative (Thread Lift)	Cannula passes too deep into the buccal fat pad Thread entrapment of Stensen's duct Oblique vectors compressing the duct against the masseter Deep passes near the mylohyoid, injuring Wharton's duct	Keep thread cannula strictly subcutaneous Avoid deep parotid or submandibular trajectories Use controlled “fan” vectors Avoid crossing buccal fat pad at depth
Postoperative	Unrecognized early duct obstruction Progressive fibrosis causing delayed symptom onset Chronic swelling misattributed to postoperative edema	Early assessment for swelling, xerostomia, gustatory pain Initiate hydration, heat, massage Refer for sialendoscopy if symptoms persist >2–3 weeks

This study has several limitations. The sample size is small, and the retrospective design limits causal inference. Additionally, the referral-based nature of a tertiary salivary gland clinic likely enriches for more severe or persistent cases, limiting conclusions regarding incidence or absolute risk. The prolonged latency between facial rejuvenation procedures and symptom onset further complicates causal attribution. Despite these limitations, this series highlights an underrecognized complication and underscores the importance of early recognition and minimally invasive management.

## Conclusion

Obstructive sialadenitis of the parotid and SMG is an uncommon and underreported complication of FRPs, distinct from sialocele and fistulas, that can significantly impact patient well-being. This study presents the largest case series to date on this topic. Surgeons performing rhytidectomy should be aware of the anatomical proximity of Stensen’s duct to the SMAS and take care to avoid traction or thermal injury near the anterior masseteric border. In cases of thread lifting, improper vectoring or deep passage may predispose to ductal kinking or scarring. Sialendoscopy is a valuable tool in the management of obstructive sialadenitis following FRPs. The minimally invasive nature of sialendoscopy and the ability to perform repeated interventions allow for effective management of complex cases while preserving gland function and patient aesthetics. [7, 23, 34] Should obstructive injury occur, prompt sialendoscopic evaluation and treatment are recommended to restore ductal patency and prevent further stasis or secondary infection. Future studies are needed in larger patient samples with long-term follow-up to validate the durability of these outcomes and refine treatment protocols for this unique patient population. [13, 35, 36]

## Declarations

### Human and Animals Rights

This study was conducted in accordance with the Declaration of Helsinki and was approved by the Institutional Review Board at Rabin Medical Center
